# Supplementary material for: General structural features that regulate integrin affinity revealed by atypical αVβ8
Source: Nat Commun. 2019 Dec 2;10:5481. doi: 10.1038/s41467-019-13248-5 (PMC6889490; doi:10.1038/s41467-019-13248-5)
Supplement: Supplementary file 1 — Supplementary Information [file 41467_2019_13248_MOESM1_ESM.pdf]

**Supplementary Information**

# General structural features that regulate integrin affinity revealed by atypical αVβ8

Wang et al.

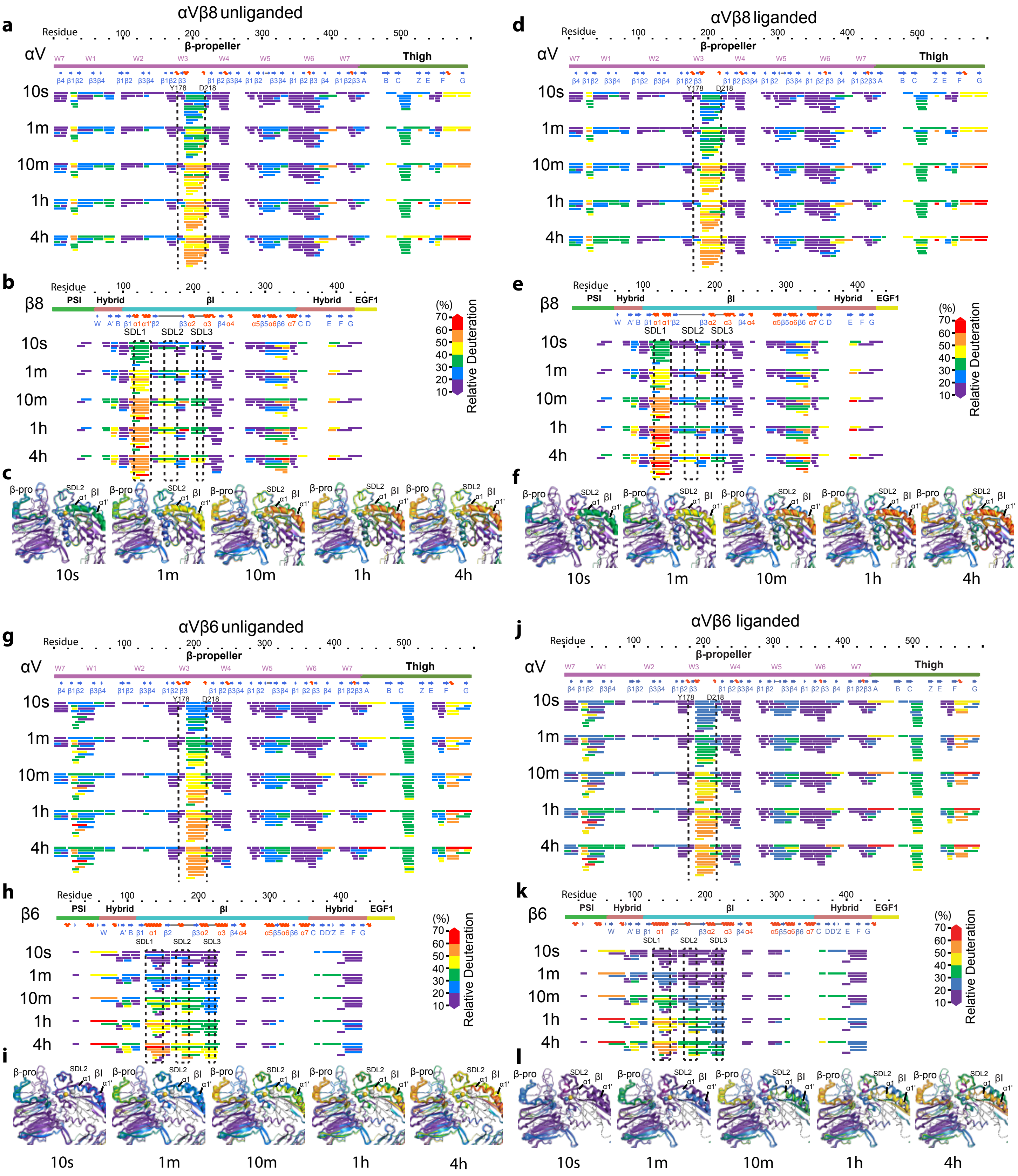


**Supplementary Figure 1. Hydrogen deuterium exchange dynamics.** (a-l). HDX of αVβ8 and αVβ6 headpiece fragments in presence or absence of TGF-β1 ligand peptide. HDX exchange on integrins with or without ligand as indicated is color-coded by sequence position (a-b, d-e, g-h, and j-k) and on the structures (c, f, i, and l) at all time points. (a-b, d-e, g-h, and j-k) show all overlapping peptides at each time point with each row showing a different set of non-overlapping peptides. (c, f, i, and l). Close-up of the β-propeller and βI domains with ribbon cartoons color-coded for each overlapping peptide except the TGF-β1 peptide is shown in magenta. Each cartoon segment is subdivided to show a different overlapping peptide. As HDX covers regions disordered in crystal structures, the structure of SDL2 from liganded αVβ8 and the hybrid domain from αVβ6 are used to model disordered regions of αVβ8; furthermore, the hybrid domain of αVβ6 swings out in presence of ligand and its position is modeled on αIIbβ3.

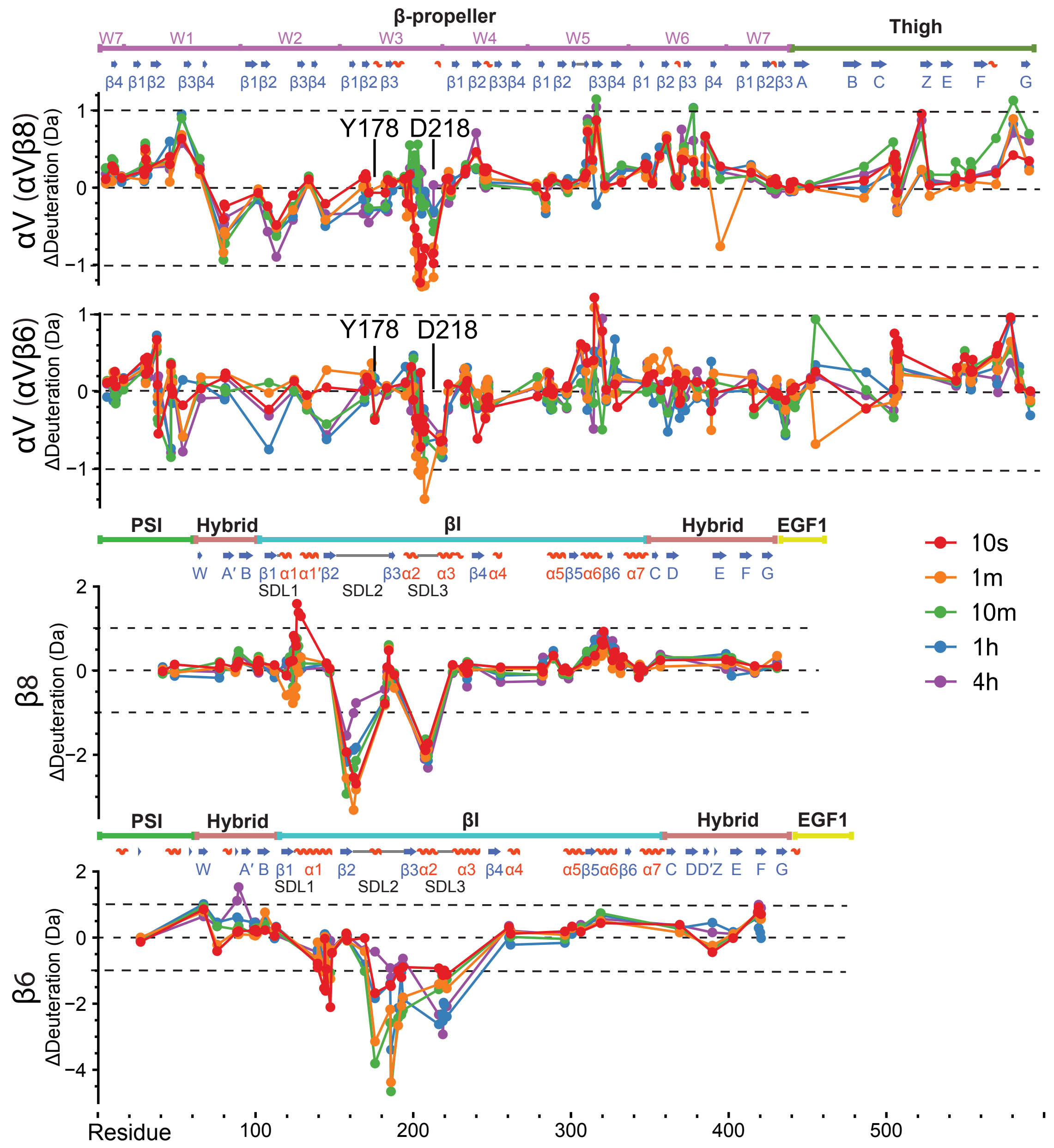


**Supplementary Figure 2. Effect of ligand binding on hydrogen deuterium exchange.** Difference in exchange for αVβ8 and αVβ6 (D incorporation for bound integrin - D incorporation for unbound integrin) is shown for each peptide plotted at the midpoint of its sequence position in the α or β-subunit. Differences greater than 1 Da (dashed lines) are considered meaningful.

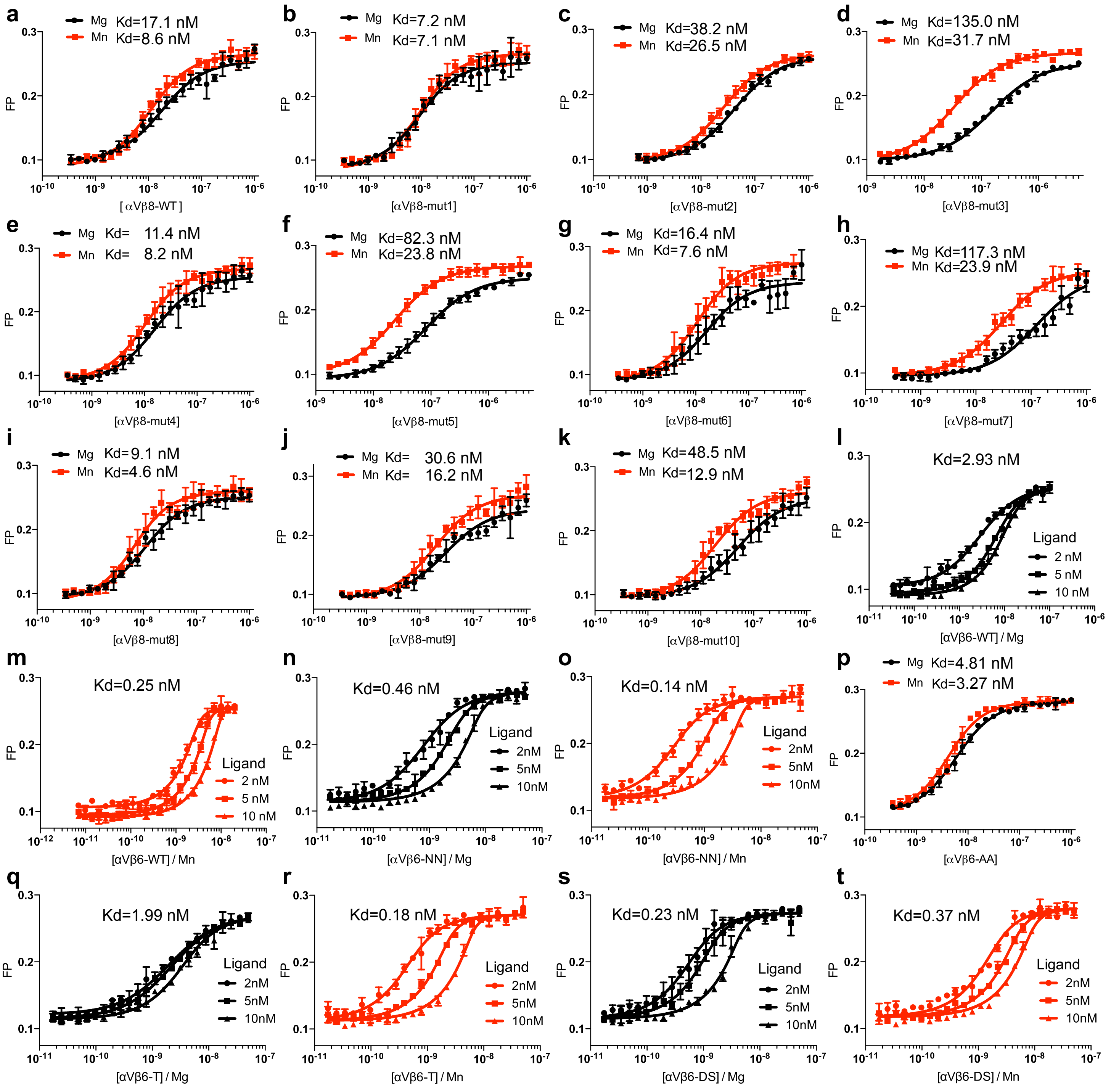


**Supplementary Figure 3. Representative affinity measurements.** (a-t). Data points and fitting curves for FP measurements. Data points were fit to a ligand-depletion model in GraphPad Prism as previously described[1]. For αVβ8 wild type and mutants, a single probe concentration was used for all measurements. For αVβ6 wild type and mutants except for αVβ6-AA, three different probe concentrations were used to more accurately determine the concentration of input ligand and better account for the effect of high-affinity binding on ligand depletion. Representative fluorescence polarization (FP) from one of two triplicate experiments (average±s.d) with fit (line) is shown for each mutant. The inset KD values were determined using the NonLinearModelFit function of Mathematica (Wolfram, Champaign IL) using all data from measurements in triplicate of two different experiments done in different months. The fit assumes that errors are independent and normally distributed and minimizes the sum of the squared errors. Source data are provided as a Source Data file.

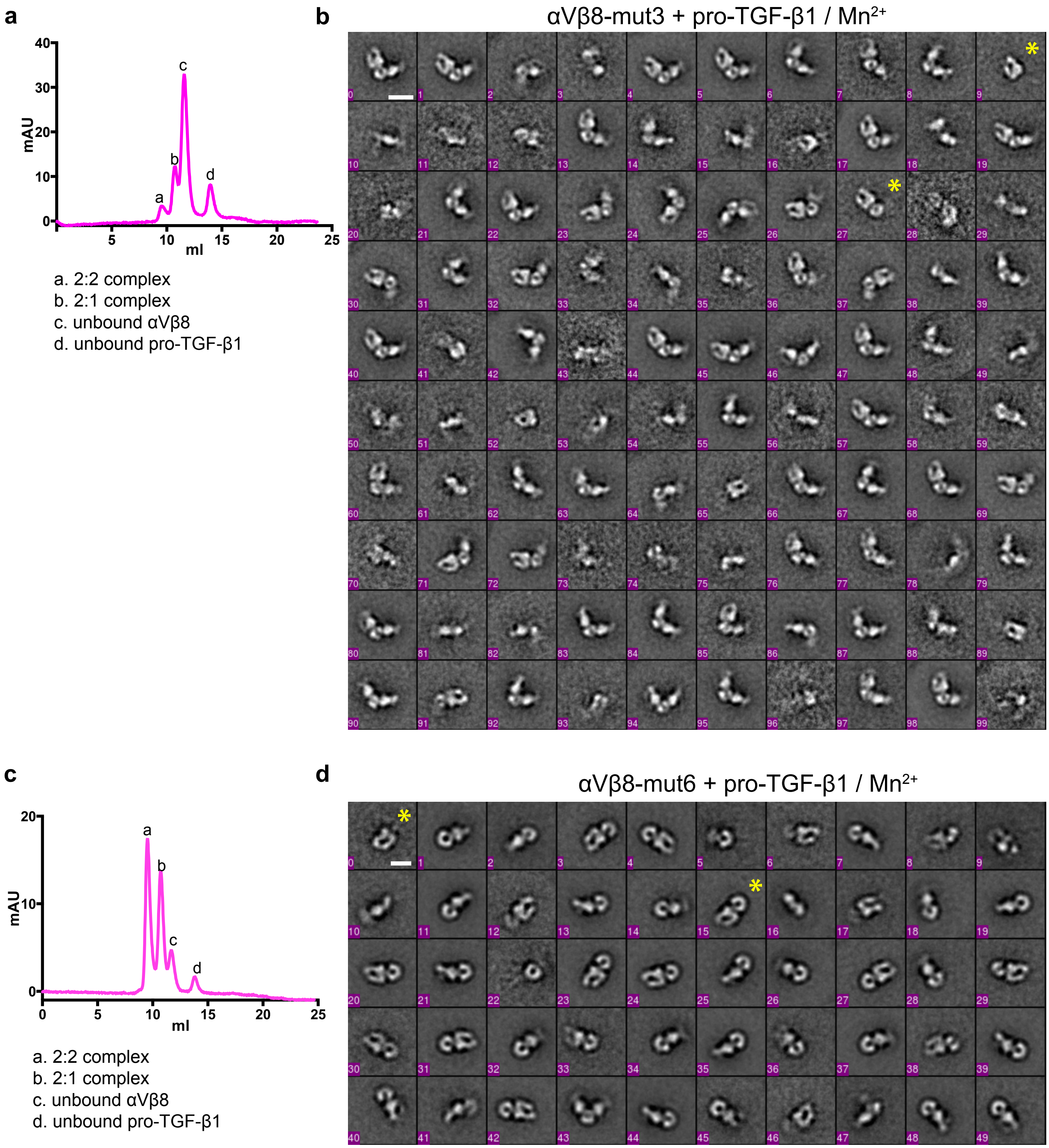


**Supplementary Figure 4. Negative stain EM class averages of mutant αVβ8 complexes with proTGF-β1.** (a). S200 gel filtration profile of 12 nM αVβ8-mut3 mixed with 8 nM pro-TGF-β1 (50 µl). (b). All class averages of αVβ8-mut3 complexed with pro-TGF-β1(5064 particles) derived from peak a in gel filtration. Class averages showing uncomplexed integrin and a 2:1 complex (asterisked) are shown in main text Figure 5. (c). S200 gel filtration profile of 12 nM αVβ8-mut6 mixed with 8 nM pro-TGF-β1 (50 µl). (d). All class averages of αVβ8-mut6 complexed with pro-TGF-β1(4823 particles) derived from peak b in gel filtration. Class averages showing uncomplexed integrin and a 2:1 complex (asterisked) are shown in main text Figure 5. The lower yield of complexes with αVβ8-mut3 (Kd 31.7 nM) is consistent with its lower affinity than αVβ8-mut6 (Kd 7.6 nM). The identity of labeled peaks (a-d) was previously established[1]. Scale bar represents 10 nm.

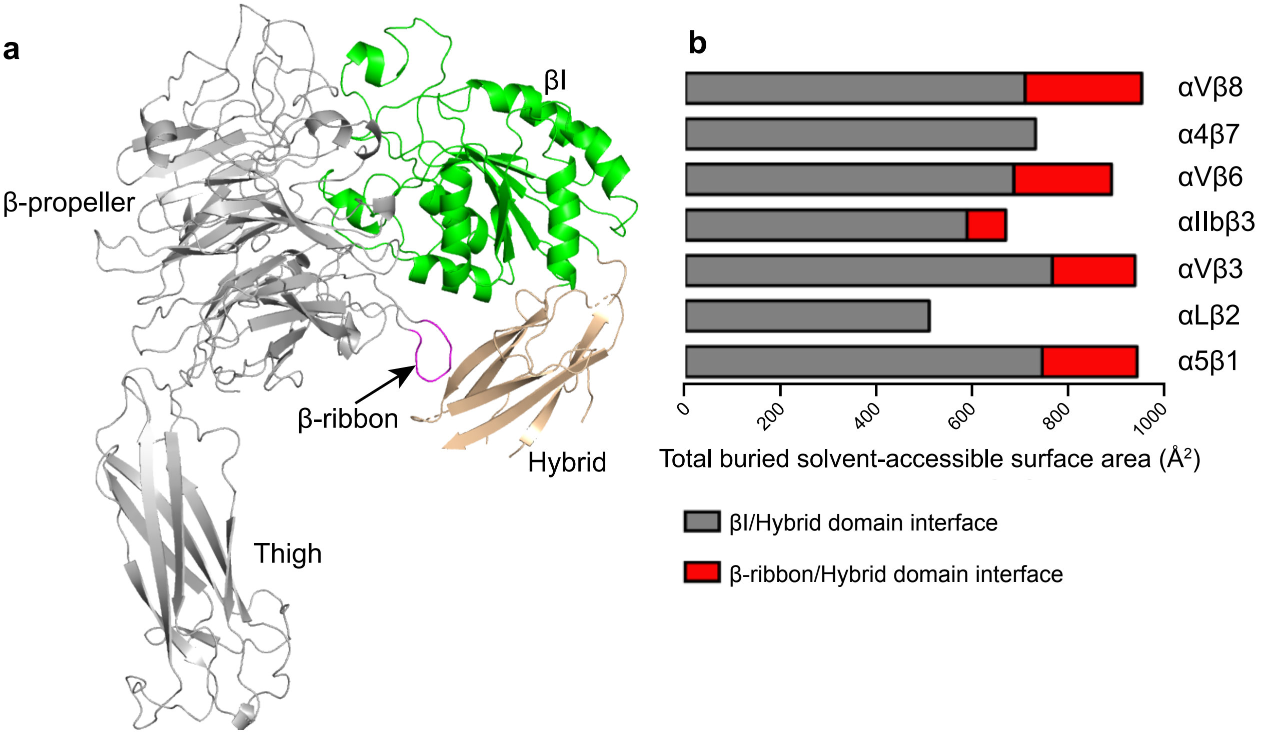


**Supplementary Figure 5. Sizes of interfaces that change in headpiece opening. (**a). Chains C and D of liganded αVβ8. (b). Buried surface area for representative integrins. All RGD-binding integrins are shown, which contain a β-ribbon extension of the β-propeller domain. Because the βI domain – hybrid domain interface alters in headpiece opening, and the β-ribbon – hybrid domain interface is broken in opening, areas of both in closed conformations are plotted. Chains C and D of liganded αVβ8 were used because there is only one unbuilt residue in the hybrid domain near the βI domain, which will have little or no effect on the area calculation. Buried areas were calculated with PISA 40.

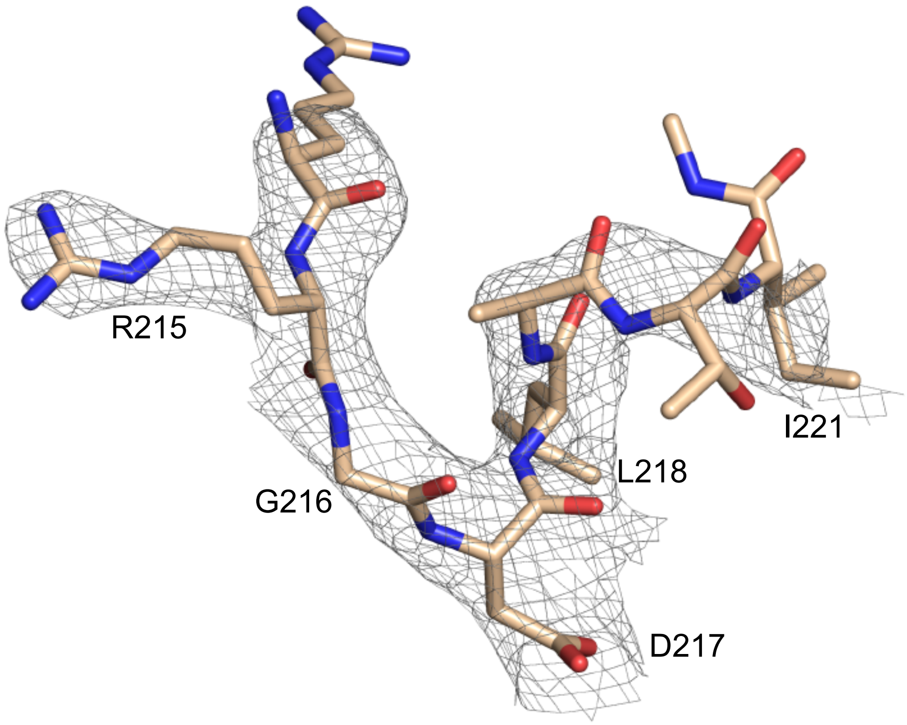


**Supplementary Figure 6.** Representative electron density. The 2Fo-Fc map contoured at 1 sigma is shown as mesh contoured within 1.7 Å around the ligand, i.e. chain F of the liganded αVβ8 structure.

**Supplementary Table 1. HDX MS Data Summary and list of experimental parameters**

| **Data Set** | αVβ6 | αVβ6 + RGD peptide | αVβ8 | αVβ8 + RGD peptide |
|---|---|---|---|---|
| **HDX reaction details** | Final $D_2O$ concentration=93.3%, $pH_{read}$=7.10, 21 °C. See also footnote a | | | |
| **HDX time course** | 10s, 1m, 10m, 1hr, 4hr | | | |
| **HDX controls** | 3 undeuterated for each condition | | | |
| **Back-exchange** | 30-35% | | | |
| **Number of peptides** | αV=156; β6=73 | αV=128; β6=47 | αV=121; β8=77 | αV=110; β8=69 |
| **Sequence coverage** | αV=88%; β6=73% | | αV=93%; β8=75% | |
| **Average peptide length Redundancy** | αV=17.8 a.a; β6=19.3 a.a Redundancy: αV=5.15; β6=4.00 | | αV=17.3 a.a; β8=17.0 a.a Redundancy: αV=3.73; β8=3.77 | |
| **Replicates (technical)** | 3 | | | |
| **Repeatability** | +/- 0.15 relative Da | | | |
| **Meaningful differences** | > 1.0 Da | | | |

[a] 15-fold dilution with labeling buffer [20mM HEPES, 150mM NaCl, 1mM $MgCl_2$ and 1mM $CaCl_2$, 99.9% $D_2O$ (pD 7.5)]. 2-fold dilution with quench buffer [4M GnHCl, 200mM sodium phosphate, 0.5M tris (2-carboxyethyl)phosphine hydrochloride (TCEP-HCl), $H_2O$], pH 2.1.

**Supplementary References**

1. Wang, J. et al. Atypical interactions of integrin αVβ8 with pro-TGF-β1. Proc Natl Acad Sci U S A 114, E4168-E4174, doi:10.1073/pnas.1705129114 (2017).
