# Supplementary material for: General structural features that regulate integrin affinity revealed by atypical αVβ8
Source: Nat Commun. 2019 Dec 2;10:5481. doi: 10.1038/s41467-019-13248-5 (PMC6889490; doi:10.1038/s41467-019-13248-5)
Supplement: Supplementary file 3 — Reporting Summary [file 41467_2019_13248_MOESM3_ESM.pdf]

nature research

Corresponding author(s): Timothy A. Springer

Last updated by author(s): October 10, 2019

Reporting Summary

Nature Research wishes to improve the reproducibility of the work that we publish. This form provides structure for consistency and transparency in reporting. For further information on Nature Research policies, see Authors & Referees and the Editorial Policy Checklist .

Statistics

For all statistical analyses, confirm that the following items are present in the figure legend, table legend, main text, or Methods section.

n/a	Confirmed	
☐	☒	The exact sample size (n) for each experimental group/condition, given as a discrete number and unit of measurement
☐	☒	A statement on whether measurements were taken from distinct samples or whether the same sample was measured repeatedly
☐	☒	The statistical test(s) used AND whether they are one- or two-sided<br>*Only common tests should be described solely by name; describe more complex techniques in the Methods section.*
☒	☐	A description of all covariates tested
☒	☐	A description of any assumptions or corrections, such as tests of normality and adjustment for multiple comparisons
☒	☐	A full description of the statistical parameters including central tendency (e.g. means) or other basic estimates (e.g. regression coefficient) AND variation (e.g. standard deviation) or associated estimates of uncertainty (e.g. confidence intervals)
☐	☒	For null hypothesis testing, the test statistic (e.g. F, t, r) with confidence intervals, effect sizes, degrees of freedom and P value noted<br>*Give P values as exact values whenever suitable.*
☒	☐	For Bayesian analysis, information on the choice of priors and Markov chain Monte Carlo settings
☒	☐	For hierarchical and complex designs, identification of the appropriate level for tests and full reporting of outcomes
☒	☐	Estimates of effect sizes (e.g. Cohen's d, Pearson's r), indicating how they were calculated

Our web collection on statistics for biologists contains articles on many of the points above.

Software and code

Policy information about availability of computer code

Data collection	Diffraction data sets were collected at the GM/CA beamline, APS, Argonne National Laboratory.
Data analysis	XDS (version 0.64);<br>Phenix (version 1.8.6);<br>Coot (version 0.8.7);<br>Pymol (version 2.2.0);<br>CCP4 (version7.0);<br>MolProbity server v.4.4 (http://molprobity.biochem.duke.edu/; queried in 2018);<br>GraphPad Prim 6;<br>DynamX (Version 3.0).

For manuscripts utilizing custom algorithms or software that are central to the research but not yet described in published literature, software must be made available to editors/reviewers. We strongly encourage code deposition in a community repository (e.g. GitHub). See the Nature Research guidelines for submitting code & software for further information.

Data

Policy information about availability of data

All manuscripts must include a data availability statement. This statement should provide the following information, where applicable:

- Accession codes, unique identifiers, or web links for publicly available datasets
- A list of figures that have associated raw data
- A description of any restrictions on data availability

Structural data have been deposited in the Protein Data Bank under accession numbers 6OM1 for unliganded αVβ8 and 6OM2 for liganded αVβ8.
Data will be released upon publication. The HDX raw datasets have been submitted to PRIDE with accession number PXD014348. Raw HDX MS data have been have been deposited to the ProteomeXchange Consortium via the PRIDE partner repository 38 with the dataset identifier PXD014348. Source data and fit values have

been provided for Figure 7 panels a and b and Supplementary Figure 3 panels a-t. Each panel's data appears as a tab in an excel file.

Field-specific reporting

Please select the one below that is the best fit for your research. If you are not sure, read the appropriate sections before making your selection.

- [x] Life sciences
- [ ] Behavioural & social sciences
- [ ] Ecological, evolutionary & environmental sciences

For a reference copy of the document with all sections, see nature.com/documents/nr-reporting-summary-flat.pdf

Life sciences study design

All studies must disclose on these points even when the disclosure is negative.

Sample size	No statistical methods were applied to determine sample size. For fluorescent polarization affinity measurements, each sample has 24 data points, which is sufficient to fit the binding curve. Each point was in triplicate and the whole experiment was repeated twice independently.
Data exclusions	No data were excluded.
Replication	All measurements were performed in triplicate.
Randomization	No randomization method was applied in this study.
Blinding	The investigators were not blinded to allocation to experiments or during outcome assessment.

Reporting for specific materials, systems and methods

We require information from authors about some types of materials, experimental systems and methods used in many studies. Here, indicate whether each material, system or method listed is relevant to your study. If you are not sure if a list item applies to your research, read the appropriate section before selecting a response.

Materials & experimental systems

n/a	Involved in the study	
[x]	[ ]	Antibodies
[ ]	[x]	Eukaryotic cell lines
[x]	[ ]	Palaeontology
[x]	[ ]	Animals and other organisms
[x]	[ ]	Human research participants
[x]	[ ]	Clinical data

Methods

n/a	Involved in the study	
[x]	[ ]	ChIP-seq
[x]	[ ]	Flow cytometry
[x]	[ ]	MRI-based neuroimaging

Eukaryotic cell lines

Policy information about cell lines

Cell line source(s)	Expi293 cells were purchased from Thermo Fisher Scientific company with catalog number A14635.
Authentication	The cell line was authenticated by the supplier.
Mycoplasma contamination	The Mycoplasma contamination test was negative.
Commonly misidentified lines (See ICLAC register)	No commonly misidentified cell lines were used.
